# Knowledge Sharing Types as Predictors of Job Performance Mediated by Problem-Solving Self-Efficacy in the Information System Integration Service Industry

**DOI:** 10.3389/fpsyg.2022.857782

**Published:** 2022-05-13

**Authors:** Jon-Chao Hong, Yi-Fang Lee, Hsin-Han Chen, Hoang Bao Ngoc Nguyen

**Affiliations:** ^1^Department of Industrial Education, National Taiwan Normal University, Taipei, Taiwan; ^2^Institute for Research Excellence in Learning Sciences, National Taiwan Normal University, Taipei, Taiwan

**Keywords:** problem solving self-efficacy, knowledge sharing, job performance, information technology, self-monitoring theory, system thinking, micro ecological system

## Abstract

Knowledge sharing is an essential approach to creative problem solving in technology firms, but few studies have considered the information system integration service industry. To address this gap, drawing on the micro-ecology theory, we developed a research model to explore the mediating role of four types of knowledge sharing (i.e., automatic response, rational reflection, ridiculed reflection, and stolen reflection) in the relationship between problem solving self-efficacy (PSSE) and IT workers’ job performance. Data were collected from 307 System Integration IT workers by using the snowball sampling method *via* a Google questionnaire. Structural equation modeling was used to test the hypotheses of the relationships between the variables. The results showed that PSSE can positively predict four knowledge sharing types; except for stolen reflection, the others can positively predict job performance. The implication of this study is that automatic response systems and rational reflection systems in knowledge sharing can enhance job performance, supported by PSSE. It is hoped that managers can generate System Integration workers’ rational reflection to effectively evoke knowledge sharing.

## Introduction

In the information industry, knowledge sharing has become an essential approach to efficiently solving technical problems (Tamjidyamcholo et al., 2013; Whitelock-Wainwright et al., 2020). According to the social exchange theory (Blau, 1964), knowledge sharing in an organization cannot only result in finding solutions efficiently, but can also lead to a cooperative tendencies. With cooperation among team members, knowledge sharing can be facilitated (Coun et al., 2019). In line with this, how to promote knowledge sharing in a team or organization to enhance employees’ performance has been studied, and a positive relationship has been found between them (e.g., Tseng and Huang, 2011; Swanson et al., 2020). Employees are required to manage knowledge flows in order to find a balance between cooperative knowledge sharing and competitive knowledge protection (Gast et al., 2019). It has been found that individual job performance is influenced by reflection upon knowledge sharing and knowledge protection (Oliveira et al., 2019). Job performance is linked with employees’ ability to achieve assigned works (June and Mahmood, 2011), that can promote the competitiveness of an information technology corporation (Pavithra and Deepak, in press). However, few studies have focused on how different types of knowledge sharing affect employees’ job performance in the information technology (IT) domain; thus, the present study focused on exploring the role of knowledge sharing in job performance.

In mental processes and output, Kahneman (2011) proposed “think fast and slow” as dual cognitive process systems: the automatic response system and the reflective thinking system. Moreover, Hong et al. (2012) applied the eight trigrams (BaGua) to explain reflective thinking in rational or logical reasoning as the Li trigram in the Chinese Yi-Ching ideology, and indicated that the Kan trigram is on the other side of reflective thinking in emotional reasoning. That is, Hong et al. (2004) divided reflection into rational reflection and affective reflection. Furthermore, according to the *I’m OK, You’re OK* theory (Harris, 2004), there are two factors which influence knowledge sharing: opportunistic and self-interested thinking (Estrada et al., 2016). Jiang et al. (2015) explained that people with an opportunistic tendency worry that their ideas will not be accepted or will be ridiculed during knowledge sharing. On the other hand, people with a self-interested tendency want to prevent their ideas from being stolen. Considering this, ridiculed reflection and stolen reflection can be included in the four types of knowledge sharing. However, Scheibehenne and von Helversen (2015) pointed out that knowledge sharing is a kind of multi-attribute decision. Some studies have focused on the factors that affect knowledge sharing, for example, leadership style (Chaudhary et al., 2021), leadership competency (Swanson et al., 2020), and organizational culture (Venkatesh et al., 2022). However, few studies have taken the four types of knowledge sharing as perspectives of organizational practice; thus, the present study extended from Kahneman’s (2011) types of system thinking to develop four processes when individuals are involved in knowledge sharing: automatic response, rational reflection, ridiculed reflection, and stolen reflection.

In line with social exchange theory (SET) (Blau, 1964; Bronfenbrenner, 1979) introduced a micro ecological system for discussing the person-process-content relationship. Individuals sharing ideas with others can improve their performance. In the information system integration service, based on the person-process-content model, employees’ problem-solving efficacy when facing a high degree of competition to solve problems in time (Cui, 2017) can prevent them from sharing or promote them to share their knowledge. Technological change increases the number of non-routine jobs and exception tasks which require more problem-solving in information technology related occupations (Reijnders and de Vries, 2018). Software engineers need to be involved in the co-alignment of processes, routines, and practices to manage product, service, and software integration with smart solutions (Huikkola et al., 2021), particularly in the information system integration service industry. Furthermore, to address the problem related to lack of engineers’ mindset of sharing knowledge in service-driven culture, this study initiated taking the engineers’ perspective of knowledge sharing to predict job performance. However, few studies have studied knowledge sharing types (Ritala et al., 2015). Drawing on SET and the micro-ecology model, the aim of this study was therefore to examine how employees’ problem-solving self-efficacy (PSSE), the four types of knowledge sharing, perceived knowledge sharing values, and job performance are all correlated within the information system integration service.

## Theoretical Background

### Problem Solving Self-Efficacy

Practically speaking, the fact that self-efficacy may have a positive effect on problem-solving efficacy has crucial implications for our understanding of the optimal ability and confidence belief in a task (Tierney and Farmer, 2004). This belief can be termed as problem solving self-efficacy (PSSE) in a special task. One’s ability and willingness to adopt strategies to monitor, adjust, and reflect upon the problem-solving process can partially influence one’s problem-solving efficacy (Caldas et al., 2016). In line with this, one’s PSSE related to information integrating jobs can be defined as ones’ confidence in and ability to solve IT problems by monitoring, adjusting, and reflecting on problem solving in that service (Whitelock-Wainwright et al., 2020).

According to the theory of social cognitive learning, PSSE can be enhanced, but it can also be inhibited (Newman et al., 2018). Cervone and Peake’s (1986) study showed that participants were led to overestimate or underestimate their self-efficacy for a special task by being provided with an irrelevant anchor. Efficacy beliefs can be easily biased as, when one faces a problem-solving task, it is difficult to correctly assess one’s abilities and skills, and the effort that is necessary to successfully carry out the task (Oh, 2020). That is, self-efficacy in problem solving is subject to that problem or phenomenon (Lau et al., 2019). Individuals have a tendency to inaccurately assess their competence, and tend to be overconfident (Carter and Dunning, 2008; Molden and Higgins, 2012). It is thus likely that when a new problem-solving task is considered as being important for one’s future work self, one’s future self-efficacy judgments will be affected by that belief (Oh, 2020). Accordingly, given the problem-solving tasks related to software functions that IT workers focus on, the role that PSSE plays was explored in this study.

### Knowledge Sharing

The knowledge sharing process is perceived to be related to sharing individual, team, and organizational knowledge. Knowledge sharing can bring significant benefits to employees and organizations due to its potential and actual advantages (Lin, 2007). In an organization, employees share knowledge with each other through storing their experiences in knowledge databases, which can lead to the improvement of their organization’s efficiency (Lin, 2006; Al Qeisi and Al Zagheer, 2015). Knowledge sharing promotes employees’ cooperation and teamwork through exchanging problem-solving experience and new ideas to improve the organization’s learning capacity (Dyer and Nobeoka, 2000; Cummings, 2014; Obrenovic et al., 2021). Regarding the influence of organizational factors on knowledge sharing, Ellahi and Mushtaq (2011) identified several factors that have an influence on knowledge-sharing behavior, namely mutual trust among staff, perceived usefulness and perceived value, job tasks, and appropriate technologies. The personality trait of conscientiousness, attitude of eagerness, and subjective norms were also found to have a positive impact on tacit knowledge sharing behavior (Obrenovic et al., 2021). According to Bock et al. (2005), four major factors that affect individuals’ attitudes toward knowledge sharing are anticipated extrinsic reward, anticipated reciprocal response, sense of self-interest, and fairness. For example, Rungsithong and Meyer (2020) found that trust is related to the effectiveness of knowledge sharing. Obrenovic et al. (2020) found that willingness to share had an impact on tacit knowledge sharing and played a mediator role between the trait of altruism and tacit knowledge sharing. Individual, organizational, and technology infrastructure are considered as factors affecting knowledge sharing behavior (Kakhki et al., 2020). Additionally, the fear of losing power due to knowledge sharing restrains young scientists (Oliveira et al., 2019), indicating that stolen reflection in knowledge sharing needs to be considered when studying types of knowledge sharing. However, few studies have considered the four types of mental processes in knowledge sharing in the information system integration service. Thus, the present study explored the levels of the four types of knowledge sharing.

### Job Performance

According to Borman et al. (1994) and Sobaih et al. (2019), job performance is the extent to which one is able to successfully carry out one’s job tasks considering the normal constraints of the job and using the available resources. It includes two types of performance, namely task or in-role performance, and contextual or extra-role performance. Also, it refers to the employees’ ability to achieve the expectations of the organization (Darvishmotevali and Ali, 2020). Borman and Motowidlo (1993) further proposed dividing job performance into task performance and contextual performance, where task performance refers to the outcome of job performance related to completing a mission within the organization, whereas contextual performance is an extra-role behavior that is not restricted by any official rules. Indeed, the behavioral and outcome aspects of performance are linked together (Sobaih et al., 2019). In line with Borman and Motowidlo’s (1993) explanation, in this study, job performance is defined as an evaluation of the individual’s job results.

According to the person-environment fit theory (Muchinsky and Monahan, 1987), if a team leader can hire employees to fit the organizational culture, the team performance can be promoted (McGuire et al., 2019). Additionally, He and Brown (2013), adopting the person-job fit theory (Edwards, 1991), studied the effect of employees’ competence on their job performance, and found that there was a positive correlation. As failure costs increase exponentially during product implementation, and trouble-shooting cycles shorten, job performance within the information technology industry becomes a competitive factor (Peiro et al., 2020; Whitelock-Wainwright et al., 2020). Thus, the level of individuals’ job performance in the information system integration service jobs was explored in this study.

## Hypotheses

### Research Model

**FIGURE 1:**
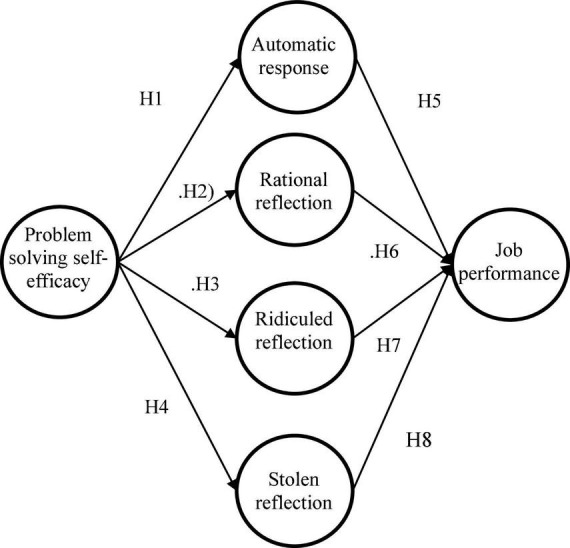
Research model.

This proposition was developed further with the micro-ecology theory (Bronfenbrenner, 1979) which addresses the relationship between person-process-content. The person-process-content model (Bronfenbrenner and Crouter, 1983) was derived from the idea of the maximization of change processes. Based on the person-process-context model, we described the variability in the developmental process as studied in this paper as a function of context (job performance), person (self-efficacy), and process (knowledge sharing). Thereby, we predicted that those employees who have powerful PSSE beliefs would be likely to dedicate resources to and share their knowledge in the problem-solving process, which would in turn result in problem-solving efficiency. When we apply this theory to work in the IT industry, the correlates between employees’ self-efficacy, knowledge sharing attitude and job performance are conceptualized as the research model for this study (Figure 1).

### Problem Solving Self-Efficacy and Knowledge Sharing

Participants usually evaluate their collaboration with partners in light of two aspects, namely the distributive fairness of the investment-reward ratio, and the procedures designed to reward contributions (Katok and Pavlov, 2013). ET has been drawn on in a number of studies to examine the multilateral relationships that are embedded in either network rational reflection or cross-organization knowledge sharing (Zhou et al., 2020). For example, it has been found that employees’ attitudes toward and intention to engage in knowledge sharing are enhanced by self-efficacy, which provides benefits for the whole organization (Chen et al., 2012). While self-efficacy has been shown to persist over time (Pajares and Graham, 1999), individuals’ judgments may change in line with changes in their perceptions of the job condition. Bandura (1986) described the connection between one’s ability to control one’s interactions and beliefs about one’s capabilities and potential to control one’s behavior (Pajares, 2002), arguing that those people who have high levels of self-efficacy tend to set themselves more challenging goals and usually work harder than those who have low levels of self-efficacy (Bandura, 1997). Some arguments arise in knowledge sharing. A previous study revealed that if employees found inequality in their knowledge sharing, they would change their attitude toward it (Hsu and Lin, 2008), but another study indicated that individual self-efficacy was more important in sharing knowledge than equity concerns (Tiedens and Linton, 2001; Bandura and Locke, 2003). However, when there is uncertainty appraisal and management is challenged in the IT industry (Brashers and Hogan, 2013), employees might not have the well-formed self-efficacy beliefs needed to be able to regulate their actions (Tamjidyamcholo et al., 2013). How employees’ PSSE relates to different types of knowledge sharing was hypothesized as follows:

Hypothesis 1. PSSE is positively related to automatic response.

Hypothesis 2. PSSE is positively related to rational reflection.

Hypothesis 3. PSSE is positively related to ridiculed reflection.

Hypothesis 4. PSSE is positively related to stolen reflection.

### Knowledge Sharing and Job Performance

Knowledge sharing is a vital social asset for organizations because it leads to better job performance and improves organizational success (Swanson et al., 2020). The Self-monitoring theory explains the tendency of individuals to manage their behaviors and expressions such that they reflect the surrounding environment (Snyder, 1974). When people have a high self-monitoring orientation, they will analyze the appropriate behavior and the best way to express their ideas in certain situations (Bell et al., 2000). For example, being able to sensitively react to situational clues and demands is an important issue for employees which results in different knowledge sharing behavior that affects the job performances in an organization (Oh and Kilduff, 2008). That is, promoting clues and demands in knowledge sharing can promote employees’ job performance in an organization (Stollberger et al., 2019; Swanson et al., 2020). Moreover, Nassif (2019) highlighted dark personalities, stating that if people are Narcissists, they will exhibit hostility and aggressiveness when their egos come under threat, while Psychopaths have been observed to worry about making negative impressions in group communications (Rauthmann, 2012). Core behavioral adaptation related to the extent to which dark traits affect one’s feeling of being worthy to share and of being competent to work (Pratim, 2007), in particular among IT professionals (Cui, 2017), has not been explored in related studies. Thus, how different types of IT employees’ knowledge sharing is related to job performance was hypothesized as follows:

Hypothesis 5. Automatic response is positively related to job performance.

Hypothesis 6. Rational reflection is positively related to job performance.

Hypothesis 7. Ridiculed reflection is positively related to job performance.

Hypothesis 8. Stolen reflection is positively related to job performance.

## Research Design

### Research Design

A questionnaire-based survey technique was used, and snowball sampling was adopted. A questionnaire form was created in Google Docs, then a message was sent to friends who work in the sector of the information system integration service in the IT industry *via* Facebook, Line, and email. They were asked to mail the form to more friends working in the same field.

In consideration of the ethical issues, we assured respondents about the anonymity of their responses (Islam et al., 2020). As both independent and dependent variables were measured at the same time, the present study took some measures to avoid the issue of common method variance (Islam and Tariq, 2018). After 2 weeks of data collection, 320 responses were returned, and after deleting incomplete questionnaires, there were 307 data available to be subjected to statistical analysis.

### Participants

Regarding the composition of the participants, 73 (23.8%) were females and 234 (76.2%) were males, indicating that the majority of employees in the information integrating service are males. In terms of age, 49 (16%) were between 21 and 30 years old, 112 (36.5%) were between 31 and 40 years old, and 132 (43%) were between 41 and 50 years old, indicating that employees aged between 41 and 50 constituted the major population of the current study.

### Research Instruments

The questionnaire items were adapted from the previously mentioned theories and research and were obtained by using the forward-backward translation method to ensure face validity. This study used the questionnaires to collect the required data and construct the content basis for the analysis. The questionnaire applied a 5-point Likert scale as the scoring model for the subsequent analysis and presentation of the results. The questionnaire reliability and validity were examined using SPSS and AMOS.

Problem solving measurement: Previous research has indicated that there is a positive relationship between creative self-efficacy and creative problem solving in the workplace (Amabile, 1996; Treffinger, 2000). We adapted the creative self-efficacy scale developed by Tierney and Farmer (2004) and embedded problem-solving self-efficacy in IT-oriented jobs into the items, for example, “I have confidence in solving IT problems” or “I can solve any problem in the information integrating system service.”

Knowledge sharing types of measurement: Automatic response and rational reflection were adapted from the dual process theory (Kahneman, 2011). The present study drew on the theory of *I’m OK, You’re OK* (Harris, 2004), and the opportunistic and self-interested orientations of knowledge sharing (Estrada et al., 2016) to design items related to ridiculed reflection and stolen reflection. Exemplary items in automatic response included: “If I find out someone made a mistake, I will point it out right away” and “I always express my suggestions immediately when they come to mind.” Rational reflection included: “If I have a new idea, I would think about whether it is logical before speaking out” and “If I have a new idea, I would compare that idea to others before I speak out.” Ridiculed reflection included: “If I have a new idea, I would think about whether it will be ridiculed before I speak out” and “If I have a new idea, I would consider whether it is too naïve before I speak out.” Stolen reflection included: “If I have a new idea, I would think about whether it will be stolen before I speak out” and “If I have a new idea, I would consider whether it will be taken as others’ new idea before I speak out.”

Job performance measurement: Given that our scale is job-specific, we had specific objectives for measuring IT job performance related to service work (Pitafi et al., 2018). That is, to assess employees’ job performance in a customer-oriented environment, we adapted Grote and Grote’s (1996) guide to performance appraisal to design our scale, including the items: “I think that by knowledge sharing with others, I can troubleshoot in the information integrating system sooner than before” and “I think that by knowledge sharing with others, I can solve more difficult problems.”

### Data Analysis

#### Item Analysis

The internal validity of each item in the constructs was examined by first-order confirmatory factor analysis (CFA) with factor loading and residual values. If the factor loading of items was lower than 0.5, the item would be deleted first, then those items with higher residual value in each construct would be deleted until the threshold values met the first-order CFA (Byrne, 2010). Table 1 indicates that after the first-order CFA, the number of items of PSSE was reduced from 6 to 4, automatic response was reduced from 6 to 4, rational reflection from 5 to 4, ridiculed reflection from 6 to 4, stolen reflection from 5 to 4, and job performance from 5 to 4.

**TABLE 1:** Item analysis.

Construct	Internal validity				External validity	
	*X*^2^/*df*	GFI	AGFI	RMSEA	FL	*t-*value
PSSE	1.835	0.994	0.970	0.052	0.690∼0.837	17.185–22.809
Automatic response	3.617	0.989	0.944	0.072	0.584 ∼0.783	13.887–19.798
Rational reflection	2.364	0.993	0.963	0.067	0.665 ∼0.841	20.724–26.651
Ridiculed reflection	1.590	0.995	0.974	0.044	0.636 ∼0.937	18.891–29.513
Stolen reflection	2.282	0.999	0.995	0.051	0.729 ∼0.896	19.095–24.379
Job performance	2.283	0.992	0.960	0.065	0.614 ∼0.861	19.801–28.756

This study evaluated the external validity of the items to determine the scope of interpretation (Cor, 2016). The value of each question responded to by all respondents was divided into the top 27% and the bottom 27%, and then a *t*-test was performed. When the *t*-value exceeds 3 (^∗∗∗^*p* < 0.001), the external validity would be considered as reaching a significant level. Table 1 shows the *t*-value of PSSE (17.185–22.809), automatic response (13.887–19.798), rational reflection (20.724–26.651), ridiculed reflection (18.891–29.513), stolen reflection (19.095–24.379), and job performance (19.801–28.756), indicating that all of the *t*-values were over 3, and met the threshold, which means that all of the items in this study had a degree of accuracy, and could be used to ascertain the degree of response for the different samples (Green et al., 2005).

#### Construct Reliability and Validity Analysis

The present study adopted Cronbach’s α to test the construct internal consistency, and composite reliability (CR) to examine the construct external consistency. Based on Hancock et al. (2018) suggestion, the values of Cronbach’s α and CR should be over 0.70. Table 2 shows that all values in each construct were above 0.70, thus meeting the threshold.

**TABLE 2:** Construct reliability and validity.

	*M*	*SD*	Cronbach’s α	CR	FL	AVE
Threshold	–	–	> 0.7	> 0.7	> 0.5	> 0.5
PSSE	3.701	0.861	0.868	0.858	0.773	0.602
Automatic response	3.135	0.947	0.810	0.807	0.713	0.514
Rational reflection	4.194	0.691	0.831	0.831	0.741	0.553
Ridiculed reflection	3.120	0.958	0.869	0.878	0.795	0.647
Stolen reflection	2.201	0.700	0.895	0.895	0.824	0.683
Job performance	4.199	0.729	0.869	0.869	0.790	0.625

Convergent validity analysis was analyzed by average variance extracted (AVE) and factor loading. Table 2 shows that the values of FL and AVE in each construct were above 0.5, which meets Hancock et al.’s (2018) suggestion and indicates that the convergent validity was acceptable in this study. Construct discriminative validity was tested by comparing two values; if the Pearson coefficient absolute values were less than the values of the AVE square root, then the discriminative power of each construct can be considered acceptable (see Table 3).

**TABLE 3:** Construct discriminative validity analysis.

	PSSE	Automatic response	Rational reflection	Ridiculed reflection	Stolen reflection	Job performance
PSSE	0.776					
Automatic response	0.478	0.717				
Rational reflection	0.314	0.096	0.744			
Ridiculed reflection	0.357	0.204	0.130	0.804		
Stolen reflection	0.349	0.109	−0.002	0.264	0.826	
Job performance	0.366	0.402	0.216	0.231	0.221	0.791

## Results

### Model Fit Analysis

Scholars have suggested that the recommended value of each fitting index is that the chi-square degree of freedom ratio (χ^2^/df) should be less than 5 (Hancock et al., 2018), while the root mean square error of approximation (RMSEA) has to be less than 0.1. It is also recommended that the goodness-of-fit index (GFI), adjusted goodness of fit index (AGFI), normed fit index (NFI), non-normed fit index (NNFI), comparative fit index (CFI), incremental fit index (IFI) and relative fit index (RFI) all exceed the threshold of 0.8 (Hancock et al., 2018), whereas the parsimonious normed fit index (PNFI) and parsimonious goodness of fit index (PGFI) are best to exceed 0.5 (Hancock et al., 2018). The statistical verification values of this study are χ^2^/*df* = 2.012, RMSEA = 0.058, GFI = 0.948, AGFI = 0.924, NFI = 0.902, NNFI = 0.903, CFI = 0.911, IFI = 0.914, RFI = 0.908, PNFI = 0.768, and PGFI = 0.730. All values reached the suggested index values and fitted the model well (Byrne, 2010). Besides, the result of a multivariate normality test showed that the Mardia’s coefficient of multivariate kurtosis was 133.911, the critical ratio of which was 25.149.

### Path Analysis

**FIGURE 2:**
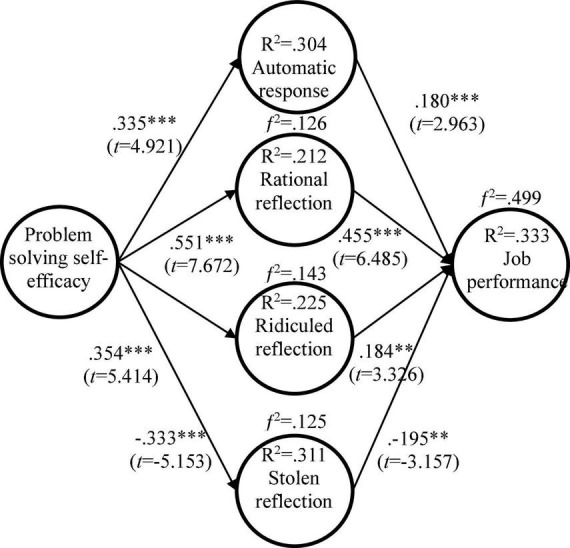
Verification of the research model. ^∗^*p*< 0.05, ^∗∗^*p*< 0.01, ^∗∗∗^*p*< 0.001.

This study adopted AMOS20 to verify the paths of the research model. Figure 2 shows that PSSE is positively related to automatic response (β = 0.335, *t* = 4.921^∗∗∗^), rational reflection (β = *0.551, t = 7.672*^∗∗∗^) and ridiculed reflection (β = *0.354, t = 5.414*^∗∗∗^), but is negatively related to stolen reflection (β = −*0.333, t = −5.153*^∗∗∗^). Figure 2 also shows that rational reflection in knowledge sharing is positively related to job performance (β = *0.455, t = 6.485*^∗∗∗^); ridiculed reflection and automatic response in knowledge sharing are positively related to job performance (β = *0.184, t = 3.326*^∗∗^;β = *0.180, t = 2.963*^∗∗^); however, stolen reflection in knowledge sharing is negatively related to job performance (β = −*0.195, t = 3.157*^∗∗^). The values of effect size (*f*^2^) between PSSE and knowledge sharing correlate, and knowledge sharing and job performance are above the threshold. The explanative power of PSSE on automatic response is 30.4 %, on rational reflection it is 21.2%, on ridiculed reflection it is 22.5%, and on stolen reflection it is 31.1%. The explanative power of the four types of knowledge sharing on job performance is 33.3%. Those effect sizes and explanative powers are all above the threshold suggested by Byrne (2010).

## Discussion

Extended from the person-process-context model, we developed a research model, self-efficacy-knowledge sharing-performance, to explore the mediating role of four types of knowledge sharing in PSSE predicting IT workers’ job performance. The hypotheses proposed in the research model were tested using AMOS, and the results are discussed as follows.

Bronfenbrenner (1979) proposed the micro ecological system to discuss the relationship between person-process-content (PPC). Bronfenbrenner and Crouter (1983) described a “person-process-context model” (PPCM) in which variability in a developmental process as studied in this paper is a function of context (job performance), person (self-efficacy), and process (knowledge sharing). According to the interaction of the PPC model, interaction between predisposing factors such as PSSE, and social behavior, such as knowledge sharing, results in specific job performance. Additionally, the self-monitoring theory explains the tendency of individuals to manage their behaviors and expressions so as to reflect the surrounding environment (Snyder, 1974). The participants’ working environment was the information system integration service industry. Therefore, we took the micro-ecology model to explore how PSSE affected their knowledge sharing tendency, and the results of this study showed that H1, H2, and H3 were positively supported, but H4 was negatively supported. Our findings are supported by the study of Hoffman and Schraw (2009) which indicated that motivational beliefs, such as self-efficacy, increase problem-solving efficiency through focused effort and strategy use. Moreover, they are consistent with another previous study which revealed that if employees found inequality in knowledge sharing, it would inhibit their attitude toward sharing knowledge with team members (Chen et al., 2012). That is, if they have a strong sense of self-efficacy in problem solving when they feel that there is inequality in knowledge sharing, they will have stolen reflection compared to the other three correlates. Conclusively, PSSE is highly related to the four types of knowledge sharing, indicating that participants who have a high level of PSSE will have positive attitudes toward sharing knowledge, but they will have lower levels of stolen reflection in sharing knowledge in the information system integration service industry.

There are many studies which have aimed to understand the relationship between knowledge sharing and job performance. For example, drawing on social capital theory, Swanson et al. (2020) found that leader competencies are critical for promoting knowledge sharing and enhancing employee job performance. In another example, based on the cultural contingency model, Venkatesh (2022) revealed that knowledge providing led to better job performance. Differing from those studies, based on social exchange theory, in the present study, we developed four types of knowledge sharing tendencies and examined their prediction of job performance. Examination of H5, H6, H7, and H8 indicated that the former three hypotheses were positively supported, but H8 was negatively supported. When core behavioral adoption is related to the extent to which one feels worthy of sharing one’s ideas (Pratim, 2007), the present study indicated that a prediction of rational reflection to job performance is higher than the other three types of knowledge sharing: automatic response, ridiculed reflection, and stolen reflection. Moreover, stolen reflection is negatively related to job performance. The results are consistent with some previous studies; for example, Nassif (2019) highlighted dark personality types, and found that people who are Narcissists will exhibit hostility and aggressiveness when their egos come under threat, while Psychopaths have been observed to worry about making negative impressions in group communications that hinder knowledge sharing and inhibit job performance (Rauthmann, 2012). Drawing on the protection motivation theory, Soral et al. (2022) found that dark personality traits enhance subordinates’ knowledge-hiding behavior and threaten their job security. In line with the dark personality types, the results of this study indicated that employees with self-interest in knowledge sharing indicated a high level of stolen reflection that led to decreased job performance. Employees in the information system integration service industry must immediately respond, so job performance and automatic response in knowledge sharing have a positive relationship.

## Conclusion

Knowledge sharing is an essential activity in an organization. Different types of knowledge sharing may result in different individual performances in the job, and this may influence organizational effectiveness. To understand this issue, the present study applied a micro ecological system to design four types of knowledge sharing, and applied AOM to design the research model. Data were collected and structural equation modeling was performed in order to test the research model hypotheses, the results of which show that PSSE is positively related to three types of knowledge sharing, but not stolen reflection. These three types of knowledge sharing were also positively related to job performance, but once again not to stolen reflection.

### Theoretical Implications

This research adapted a micro ecological system and the social exchange theory to develop four types of knowledge sharing. These types advance the understanding of knowledge sharing behavior in previous studies. In line with this, other types of knowledge sharing may be developed according to different organizational needs. Among the four types of knowledge sharing, rational reflection can most positively predict one’s job performance. With rational reflection, the proposed ideas may be more suitable for solving job problems; thus, IT managers have to encourage their employees to practice rational reflection and thinking before take an action and to increase the effectiveness of members’ knowledge sharing. Besides, IT companies need collaborative problem solving. If employees with ridiculed reflection hesitate to share their knowledge, that will hamper their job performance and their team effectiveness; thus, IT managers need to develop a friendly chatting environment to decrease the ridiculed reflection of employees.

### Practical Implication

The results of this study have at least three practical implications for the field. First, this study contributes a novel, identity-centered perspective to explain four types of knowledge sharing. Specifically, we argue that the strength with which a person identifies with that aspect of PSSE will predict that person’s willingness to engage in knowledge sharing. The impact of an individual’s PSSE should be considered in terms of how its effects alter job performance. Second, this study provides a strong example of the importance of management approaches to avoid stolen reflection tendency in knowledge sharing. Finally, the results of this study revealed that PSSE is the antecedent of job performance mediated by knowledge sharing; thus, to enhance knowledge sharing tendency, employees with high levels of PSSE are the best choice to work in the information system integration service industry.

### Limitation and Future Study

Regarding gender, most participants in this study were male because the IT industry attracts more males in Taiwan. Thus, the present study did not analyze the difference in knowledge sharing by gender. Future studies may focus on some industries which recruit more female workers, such as bank workers, to identify any gender differences in knowledge sharing and job performance. Besides, this study was conducted on a limited sample of 307 System Integration IT workers. Thus, it is essential to expand the research samples to different IT sectors in future research.

There are many types of job categories in the information system integration service industry, for example, information security, programming, and so on. Different job attributes have different needs for knowledge sharing; thus, future studies may identify each of these subcomponents’ individual impacts on knowledge sharing behavior, and may undertake a comparative study to understand the possible effects of knowledge sharing differences in different types of organizations.

Diversity researchers have long appreciated the importance of articulating effects of individual differences as an integral part of group knowledge sharing. The effect of four types of knowledge sharing: automatic response, rational reflection, ridiculed reflection, and stolen reflection should be further examined in different organizational cultures to have a greater variety of different perspectives to advance the meaning of knowledge sharing research.

To test the external validity of the questionnaire items, the present study applied *t-*tests to compare the top 27% and bottom 27% to ascertain whether the value was over 3 to ensure the external validity of each item. There are some other statistical methods, for example, the heterotrait-monotrait ratio of correlations, for examining the external validity of questionnaire items; thus, other approaches may be applied in future studies to test the item external validity.

## Data Availability Statement

Datasets are available on request.

## Author Contributions

J-CH contributed to the conceptualization, validation, investigation, resources, writing—original draft, project administration, supervision, and funding acquisition. Y-FL contributed to the writing—review and editing and methodology. H-HC contributed to the software, formal analysis, and term. HN contributed to the format editing and preparing manuscript for submitting. All authors contributed to the article and approved the submitted version.

## Conflict of Interest

The authors declare that the research was conducted in the absence of any commercial or financial relationships that could be construed as a potential conflict of interest.

## Publisher’s Note

All claims expressed in this article are solely those of the authors and do not necessarily represent those of their affiliated organizations, or those of the publisher, the editors and the reviewers. Any product that may be evaluated in this article, or claim that may be made by its manufacturer, is not guaranteed or endorsed by the publisher.
